# The Importance of the Macroscopic Geometry in Gas‐Phase Photocatalysis

**DOI:** 10.1002/advs.202105363

**Published:** 2022-03-03

**Authors:** Fabian Matter, Markus Niederberger

**Affiliations:** ^1^ Laboratory for Multifunctional Materials Department of Materials ETH Zurich Vladimir‐Prelog‐Weg 5 Zurich 8093 Switzerland

**Keywords:** aerogels, monoliths, nanoparticles, photocatalysis, photoreactors

## Abstract

Photocatalysis has the potential to make a major technological contribution to solving pressing environmental and energy problems. There are many strategies for improving photocatalysts, such as tuning the composition to optimize visible light absorption, charge separation, and surface chemistry, ensuring high crystallinity, and controlling particle size and shape to increase overall surface area and exploit the reactivity of individual crystal facets. These processes mainly affect the nanoscale and are therefore summarized as nanostructuring. In comparison, microstructuring is performed on a larger size scale and is mainly concerned with particle assembly and thin film preparation. Interestingly, most structuring efforts stop at this point, and there are very few examples of geometry optimization on a millimeter or even centimeter scale. However, the recent work on nanoparticle‐based aerogel monoliths has shown that this size range also offers great potential for improving the photocatalytic performance of materials, especially when the macroscopic geometry of the monolith is matched to the design of the photoreactor. This review article is dedicated to this aspect and addresses some issues and open questions that arise when working with macroscopically large photocatalysts. Guidelines are provided that could help develop novel and efficient photocatalysts with a truly 3D architecture.

## Graphical abstract

The use of macroscopic monolithic photocatalysts holds great potential for technological exploitation if the geometry of the photocatalyst is optimally adapted to that of the photoreactor. This review article is devoted to this aspect and addresses some problems and open questions that could contribute to the development of novel and efficient photocatalysts with a truly 3D architecture.

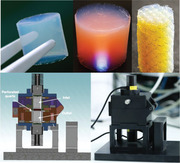

## 1 Introduction

The conversion of solar energy into chemical energy is expected to make a significant contribution to solving some of the most pressing energy and environmental problems of our time. Photocatalysis plays a unique role in this context, using only light to enable a number of important reactions in the fields of solar fuel generation, environmental remediation (e.g., wastewater treatment and air purification), and in organic and polymer synthesis.^[^ ^1^, ^2^, ^3^, ^4^, ^5^, ^6^ ^]^ The great advantage of photocatalysis is that with the help of light as the sole energy source, pollutants in air and water, for example, can be broken down or important chemical reactions such as water splitting, CO_2_ reduction or nitrogen fixation can be carried out. The spectrum of photocatalysts and photocatalytic systems developed is similarly broad as the potential applications, ranging from powders with a simple composition (e.g., titania) to relatively complex configurations combining a variety of materials (e.g., Z‐schemes and hybrid photocatalysts).

Although some applications of photocatalysis work excellently and have already been commercialized (e.g., the use of TiO_2_ in self‐cleaning window glasses),^[^ ^7^ ^]^ significant progress still needs to be made, especially in the area of solar fuels, clearly one of the most important topics, to become technologically relevant. Among the many properties of a photocatalyst that need improvement, two stand out: the ability to form electron‐hole pairs under visible light and the prevention of recombination of these electron‐hole pairs. But of course there are other aspects that must be fulfilled for efficient photocatalysis, such as large surface areas for good interaction between reactants and photocatalyst, and suitable surface chemistry that provides both optimal adsorption of reactants and desorption of products, as well as selectivity where necessary.^[^ ^8^ ^]^ Undoubtedly, these are many parameters that require a holistic approach, ideally taking into account aspects at the level of photocatalyst composition, crystal structure, morphology, or even considering combinations of several materials. At the compositional stage, mainly bandgap engineering is performed, i.e., the composition is optimized so that the semiconductor photocatalyst absorbs visible light. In terms of crystal structure, the main aim is to achieve high crystallinity, combined with the possibility of generating particularly active phases. Morphological control mainly includes the ability to synthesize an optimal particle size, ideally in combination with a particle shape that exposes crystal facets that are particularly reactive.^[^ ^9^ ^]^ The field of material combinations offers so many possibilities that it is beyond the scope of this paper to mention them all. It ranges from heterojunctions between semiconductors with suitable band alignments to the use of semiconductors with surface‐adsorbed molecular complexes, which in themselves offer an immense variety of photocatalytic and optical properties. Which of these strategies is ultimately followed depends on the form in which the photocatalyst is used in the reaction. Most photocatalytic reactions today are carried out with powders and films predominantly in liquid media. In terms of optimization of composition and crystal structures, there are no major differences between powders and films, i.e., a suitable composition and crystal structure will perform comparably well in powders and films, and the positive effect of exposing particularly reactive crystal facets is also beneficial in both cases. However, in terms of morphological control, there are some differences. Particle size is an important parameter especially for powders, while surface roughness, porosity and film thickness are more crucial for films. There are also differences in the choice of possible material combinations. In powders, combining solids with molecular components to form hybrid photocatalysts^[^ ^10^ ^]^ is more promising than forming heterostructures, which are easy to fabricate in films but difficult to realize in powders. Regardless of the shape of the photocatalyst and regardless of which parameter is optimized, the improvements take place between the atomic (composition and structure) and the nanoscale level (particle size, heterostructure formation). If we now include control over particle agglomeration, particle accumulation and superstructure formation, as well as film microstructure, we end up on the scale of a few hundred micrometers at most. In other words, the optimization of photocatalysts is mainly done via nano‐ and microstructuring, completely neglecting larger scales, so there is no such thing as “macrostructuring”. This makes sense, of course, because the critical properties that determine the photocatalytic activity of powders and films are mainly defined by nano‐ and microscale features. However, if we now move to photocatalysts that are macroscopically large, not just in two dimensions like the films, but in all three dimensions, then macrostructuring, i.e., the tuning of the macroscopic geometry, becomes important. This is the case, for example, with nanoparticle‐based aerogels. These relatively novel structures combine nanoscale properties (e.g., large surface areas, quantum size effects) of the constituent nanoparticulate building blocks with a macroscopic body.^[^ ^11^, ^12^, ^13^ ^]^ When such aerogels are used in the form of monoliths in photocatalysis, completely new possibilities open up. In addition to composition, crystal structure, morphology and material combinations, which also play a role in aerogels, macroscopic geometry now comes into play as an additional optimization parameter. This aspect has only recently been increasingly taken up and forms the basis for our review article. Not only do we see immense potential here to further improve the efficiency of photocatalysts (without necessarily changing their composition), but many exciting new scientific questions arise when using such truly 3D photocatalysts. For example, how does a reactant move through such a porous network or how does light interact with aerogels to allow photocatalytic reactions to occur at all? The knowledge in this direction then allows a systematic optimization of the geometry, which, as our results have shown, allows a significant improvement of the photocatalytic performance of the same material.

In this review we address some of these challenges, focusing on our recent work on this topic, and we offer guidance to further improve the geometric aspects of macroscopically sized photocatalysts. We begin with a brief literature review of previous efforts to fabricate 3D structured photocatalysts before moving on to discuss aspects of illumination and gas flow using nanoparticle‐based aerogel monoliths. We also present preliminary ideas regarding optimization of the macroscopic geometry of such aerogels and we make suggestions on how the optimal design of a gas‐phase flow reactor for photocatalysis might look like.

## 2 Geometry in the Literature

Optimization of macroscopic geometry is fundamental in classical catalysis. One example with a multi‐billion dollar market are catalysts for vehicle exhaust. They reduce the emission of pollutants contained in the exhaust gases of a car's internal combustion engine. The working principle of exhaust gas converters is based on heterogeneous catalysis running on a monolithic ceramic structure with a honeycomb‐like architecture as a support for noble metals that promote the desired chemical reactions at lower temperatures. The honeycomb structure provides a large surface area for optimum contact between the flowing exhaust gases and the catalyst surface, which is essential for high reaction rates. It also facilitates gas flow through the catalyst and controls possible pressure losses.^[^ ^14^ ^]^ The technologically extraordinarily successful example of automotive exhaust catalysts suggests that ceramic monoliths with large surface area and high porosity, in combination with noble metals, could also be of great value for photocatalysis.

**Figure 1:**
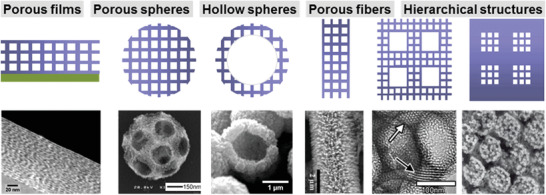
Different 3D‐titania morphologies illustrated schematically with representative electron micrographs. Reproduced with permission.^[^ ^21^ ^]^ Copyright 2014, American Chemical Society.

Bridging length scales is a fundamental principle of materials science that has been applied in particle synthesis, assembly, and processing as well as in the development and improvement of photocatalysts.^[^ ^15^, ^16^ ^]^ Already in 2008, Aprile et al. summarized the physical approaches to enhance the photocatalytic activity of titanium dioxide by spatial structuring of the semiconductor at different length scales from subnanometer to submillimeter.^[^ ^17^ ^]^ Examples covering this size scale range from nanoparticles to clusters, mesoporous films, ordered arrays of nanotubes, inverse opals, or photonic sponges.^[^ ^18^, ^19^, ^20^, ^21^ ^]^ Although these structures can reach macroscopic sizes in one (e.g., in electrospun fibers) or two dimensions (e.g., films), at least one dimension remains limited to the submillimeter range. This limitation is also evident in the review article by Fattakhova‐Rohlfing et al. dedicated to the architectures of titanium dioxide, the most commonly used photocatalyst.^[^ ^21^ ^]^ Although nanostructured titanium dioxide can assume many different shapes and morphologies (**Figure** **1**), they are never present as macroscopic bodies extending over several mm or even cm. The situation is similar for 2D and 3D ordered macroporous materials and photonic crystals, which are extensively investigated for applications in photocatalysis due to their large surface areas and fast mass transport.^[^ ^22^, ^23^ ^]^ Again, the geometrical aspects are limited to the pore size and their arrangement in 2 and 3 dimensions. Although the films can reach sizes of several cm^2^, the macroscopic nature is not decisive for the photocatalytic performance, but the microstructure of the pores. Analogous to the dimensionality of pores, geometrical aspects of photocatalytically active nanostructures are mainly discussed in the context of particle morphology. There are many reviews on 1D and 2D photocatalysts,^[^ ^24^, ^25^, ^26^, ^27^, ^28^, ^29^ ^]^ but even if the review explicitly deals with 3D architectures, the geometrical aspects are mostly limited to the level of particles or particle assemblies.^[^ ^30^ ^]^ In fact, however, there are options to advance into macroscopic size domains, for example through additive manufacturing or self‐assembly processes (with or without templating). We will address this topic again in more detail below. In any case, the literature on truly 3D photocatalysts is still very limited. On the one hand, this is certainly due to the difficulties of producing such large structures from nanoscale building blocks,^[^ ^16^ ^]^ and on the other hand, monolithic photocatalysts do not appear very advantageous at first glance. In particular, their handling and the design of suitable photoreactors are not trivial (see Section 6). However, we are convinced that the study of monolithic structures, especially the optimization of their geometry as well as the development of photoreactors suitable for them, holds enormous and as yet unexploited potential for the further development of photocatalysis.

**Figure 2:**
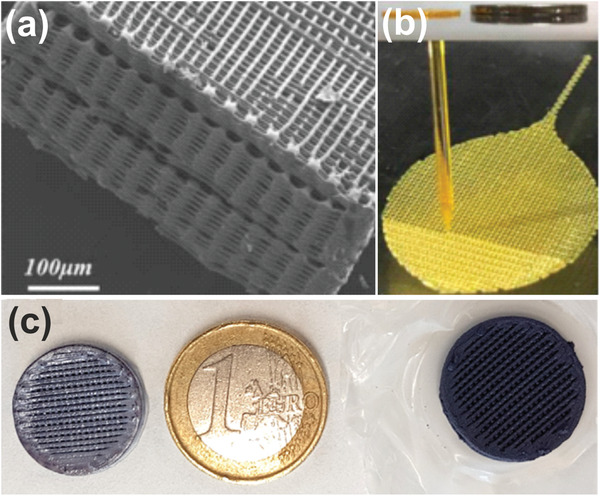
a) Scanning electron microscopy (SEM) image and b) optical image of a 3D printed artificial leaf after calcination. Reproduced with permission.^[^ ^34^ ^]^Copyright 2018, American Chemical Society. c) Photographs of two 3D printed TiO_2_ monoliths composed of TiO_2_ particles that were impregnated with Au nanoparticles after (left) and before the printing process (right). Reproduced with permission.^[^ ^35^ ^]^ Copyright 2018, American Chemical Society.

Among the emerging methods for the synthesis of macroscopic photocatalysts, colloidal gelation^[^ ^31^ ^]^ and printing approaches^[^ ^32^ ^]^ are particularly versatile in terms of control over the macroscopic geometry. Therefore, we will focus on these two methods in our brief literature overview. There are some fundamental differences between these two methods, but also some similarities depending on which printing technique is used. Colloidal gelation describes the self‐assembly of colloidal particles into volume‐filling gels of macroscopic size, typically using neither templates nor substrates. This is in sharp contrast to most printing processes, in which a ceramic, metallic or polymer substrate is first printed, onto which the photocatalytic material is then applied in a second step using classical coating processes. However, there are also direct printing techniques such as inkjet printing that allow the deposition of photocatalysts that are potentially self‐supporting and thus do not need a substrate or scaffold. In inkjet printing, the photocatalyst, mostly a semiconductor, is deposited onto a substrate using a colloidal dispersion that serves as the ink. The use of colloidal dispersions as starting materials leads to similarities between colloidal gelation and inkjet printing. However, in colloidal gelation, the nanoparticulate building blocks are usually fully formed in the dispersion, whereas in inkjet printing precursor sols are sometimes used and the formation of the final material occurs in the printed structure upon annealing. In colloidal gelation, one tries to avoid an annealing step at the end to keep the gel structure as unchanged as possible. Nevertheless, in both methods it is advantageous to start with inks or dispersions containing fully formed and crystalline nanoparticles, because in this way it is possible to completely separate particle synthesis from particle assembly/processing. This eliminates the problem of having to adapt the experimental conditions of particle synthesis to those of the assembly process, and the entire repertoire of synthesis methods for photocatalytic particles can be used, providing many opportunities to control parameters important for photocatalytic activity, such as composition, crystal structure, and particle morphology. In addition, different types of nanoparticles can be prepared separately and then combined in a dispersion in the desired ratio. The use of such complex mixtures of prefabricated nanoparticles provides access to photocatalysts that have properties precisely tailored to specific reactions. In particular, the combination of semiconductors with noble metals is widely used in photocatalysis.^[^ ^33^ ^]^ However, it should also be mentioned that the preparation of suitable dispersions and inks can be challenging because, depending on the synthesis history, the particles may be difficult to disperse in the solvents needed for gelation or printing. For example, particles synthesized in hydrophobic solvents in the presence of surfactants to suppress agglomeration are not easily dispersed in hydrophilic media. This difficulty is, of course, increased when particles from different syntheses are combined in the same solvent. Stable dispersions and suitable ink formulations with the desired particle concentrations and rheological properties can only be produced if the surface chemistry of the nanoparticles is known and can also be specifically optimized for gelation or for the printing process. As an illustrative example of the capabilities of inkjet printing of 3D photocatalysts, **Figure** **2** shows an artificial microleaf with a macroporous architecture comparable to those of natural leaves.^[^ ^34^ ^]^ The authors used a carefully formulated TiO_2_‐based sol‐gel ink to achieve suitable rheological properties. The excellent mass transfer capability of such an architecture was demonstrated by CO_2_ reduction in the gas phase, where the leaves exhibited a 2–6‐fold increase in gas evolution compared to the corresponding powders. Another example of a 3D printed TiO_2_ monolith impregnated with gold nanoparticles (Figure 2c) shows a microfilament structure that is highly active for the photocatalytic generation of hydrogen from a water/ethanol mixture in the gas phase.^[^ ^35^ ^]^ The photocatalytic activity could be tuned by controlling the size of the microfilaments.

Aerogels with their large surface area and extensive porosity seem to be predestined for photocatalysis.^[^ ^36^ ^]^ They can be easily prepared as macroscopically large monoliths and are thus true 3D materials. In principle, aerogels are accessible by additive manufacturing,^[^ ^37^, ^38^ ^]^ but compared to colloidal gelation, where particles are arranged almost simultaneously, uniformly and within a few seconds to minutes from millimeters to centimeters in all three spatial directions, inkjet printing is a layer‐by‐layer and relatively slow process. Since colloidal gelation results in volume‐filling gels, the geometric shape of the monolith is determined by the mold in which the gelation is performed. However, compared to inkjet printing, it is difficult to control the internal structure of the aerogels (as was done, for example, beautifully in the microleaf in Figure 2a). The synthesis of the aerogels is reproducible, but in comparison to mesoporous materials or inverse opals the pore size, pore size distribution and their orientation, but also the local placement of the nanoparticulate building blocks cannot be precisely controlled.

**Figure 3:**
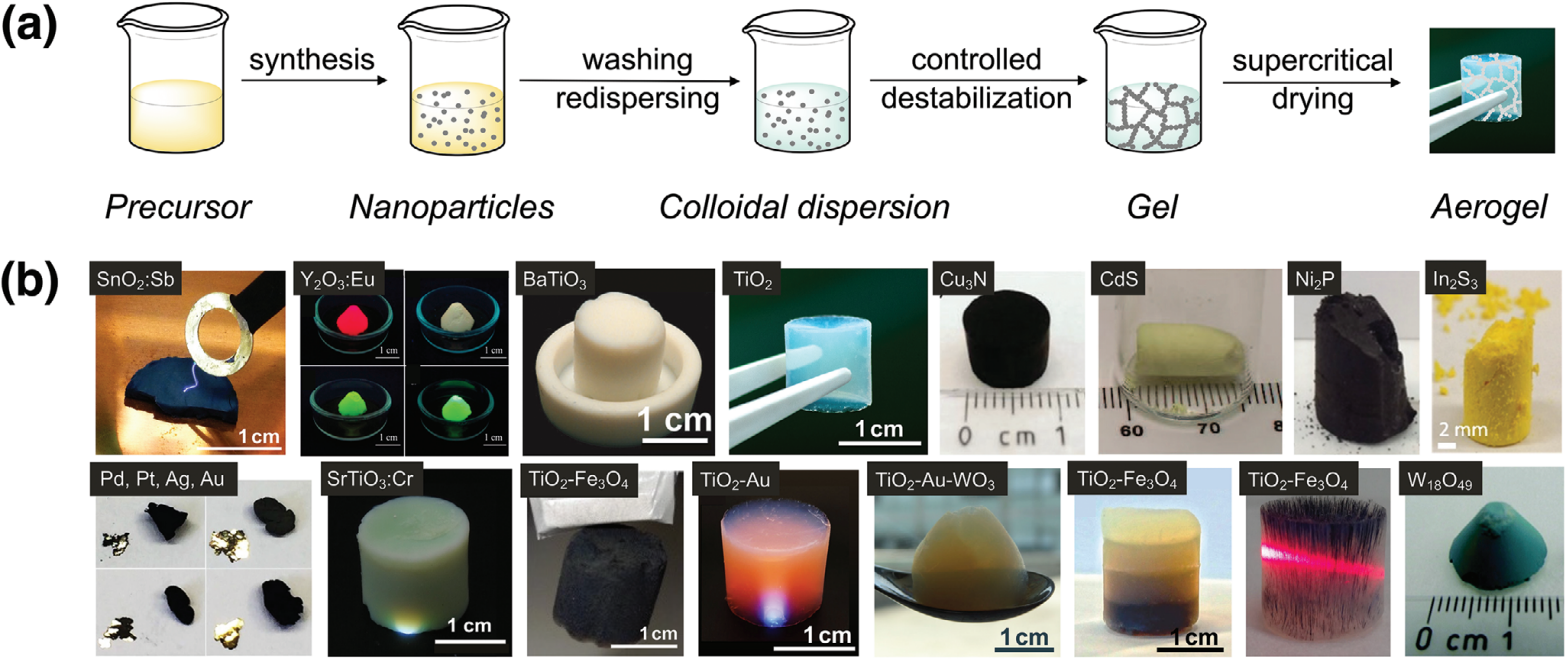
a) Schematic of the preparation of nanoparticle‐based aerogels from the synthesis of the nanoparticles to their dispersion, gelation and supercritical drying. Reproduced under the terms of the Creative Commons Attribution License CC BY 4‐0.^[^ ^44^ ^]^ Copyright 2021, The Authors, publisched by Swiss Chemical Society. b) Examples of single‐ and multi‐component nanoparticle‐based aerogels obtained by gelation of metal, metal oxide, metal nitride, metal phosphide, and metal chalcogenide nanoparticles. Reproduced with permission: SnO_2_:Sb (Copyright 2014, Royal Society of Chemistry),^[^ ^45^ ^]^ Y_2_O_3_ (Copyright 2016, American Chemical Society),^[^ ^46^ ^]^ BaTiO_3_ (Copyright 2014, Wiley‐VCH),^[^ ^47^ ^]^ TiO_2_ (Copyright 2020, Elsevier),^[^ ^31^ ^]^ Cu_3_N (Copyright 2016, Royal Society of Chemistry),^[^ ^48^ ^]^ CdS (Copyright 2005, American Association for the Advancement of Science),^[^ ^39^ ^]^ Ni_2_P (Copyright 2014, American Chemical Society),^[^ ^49^ ^]^ In_2_S_3_ (Copyright 2018, American Chemical Society),^[^ ^50^ ^]^ Pd, Pt, Ag, Au (Copyright 2019, American Association for the Advancement of Science),^[^ ^51^ ^]^ SrTiO_3_ (Copyright 2017, Royal Society of Chemistry),^[^ ^52^ ^]^ TiO_2_‐Fe_3_O_4_ (Copyright 2014, Royal Society of Chemistry),^[^ ^53^ ^]^ TiO_2_‐Au (Copyright 2017, Royal Society of Chemistry),^[^ ^54^ ^]^ TiO_2_‐Au‐WO_3_ (Copyright 2014, American Chemical Society),^[^ ^55^ ^]^ W_18_O_49_ (Copyright 2016, Royal Society of Chemistry).^[^ ^56^ ^]^

The preparation of aerogels by the gelation of colloidal nanoparticle dispersions is summarized in **Figure** **3**a. The synthesis of nanoparticle‐based aerogels has extensively been reviewed.^[^ ^11^, ^12^, ^31^ ^]^ Depending on the gelling mold or on the type of scaffold used (see Section 5), different macroscopic geometries are accessible. After a solvent‐exchange process, the gels are supercritically dried, resulting in aerogel monoliths with macroscopic sizes. A unique feature of nanoparticle‐based aerogels is that the properties of the nanoscale building blocks do not change during the fabrication of the aerogels and are thus preserved in the final macroscopic body. These are, on the one hand, the large surface areas and, on the other hand, the size‐dependent properties that are typical for semiconductor nanocrystals.^[^ ^39^, ^40^ ^]^ This in turn means that the properties of the aerogel monoliths can be precisely tailored by the choice of the starting nanoparticles. Therefore, it is important to carefully select the building blocks with respect to a targeted application, both in terms of their composition and their crystallinity, size, shape and surface chemistry. All these parameters influence the physico‐chemical properties as well as the structure and morphology of the aerogels. After more than 15 years of research, a large variety of nanoparticle‐based aerogels are readily available. Although the aerogels still suffer from varying quality of the monolithic structure, they offer an enormously wide range of functional properties that go far beyond photocatalytic activity, such as ferroelectric, luminescent, magnetic, electrocatalytic or electrically conductive properties (Figure 3b).^[^ ^11^, ^12^, ^13^ ^]^ At the same time, the examples in which such aerogel monoliths are used directly in photocatalysis without further processing are very rare. Typically, the monoliths are immersed in the reaction solution in which photocatalysis takes place and where they then break down into smaller fragments, or they are processed into a slurry and deposited as thin films on a (conductive) substrate. Thin film fabrication is particularly common in photoelectrochemical applications, where electrodes are required.^[^ ^41^, ^42^, ^43^ ^]^ It is obvious that in all the cases where the monolith is further processed, its shape does not matter and therefore its geometry is not exploited.

Monolithic structures have a whole range of characteristic features that do not occur in this form in powders and films. In particular, they are not easy to manufacture. It takes some experience in synthesizing these materials to get them of the quality needed for photocatalysis. This includes optical translucency, which plays a key role for efficient illumination. It is lost when the monolith falls apart on contact with a liquid or when it is pulverized. That is why it is advantageous to actually use the aerogels in its original monolithic form, which, however, is only possible if the chemical reaction takes place in the gas phase, which in turn necessitates the development of special reactors. At the same time, working in the gas phase also has very specific advantages over liquid phase photocatalysis beyond the stability of the monolith, such as easy separation of the photocatalyst and the product (if liquid) from the reaction medium, fast mass transport compared to liquid environment, less corrosion of the semiconductor photocatalyst, e.g., due to harsh pH conditions, no light absorption by the solvent, and no limitations related to solubilities of reactants and products, which is a disadvantage especially when using gaseous compounds in liquids.

With their nanoscale properties, high porosity and possible light transmittance, aerogel monoliths offer interesting opportunities not only to study the influence of macroscopic geometry on photocatalytic activity, especially in comparison with thin film and powder samples, but also to investigate mass and photon transfer in complex 3D structures.^[^ ^32^ ^]^ Once these aspects are understood, it will be possible to improve photocatalysts not only at the material level and on the size scale from nm to µm, but also at the 3D level, as has long been done successfully in classical catalysis. In the following, we will elaborate on some of these points, building heavily on our recent findings.

## 3 Geometry and Illumination

The illumination, i.e., the light input by the lamp as well as the light absorption properties of the photocatalyst determine how many photons reach the sample and how many charge carriers are available for subsequent processes. Accordingly, light management is fundamental to achieve high photocatalytic activity.^[^ ^57^ ^]^ Light manipulation includes the control of optical reflectance, scattering, transmittance, and absorption, and can encompass the optimization of material composition as well as modification of its morphology, e.g., in the form of patterned micro‐ and nanostructures.^[^ ^58^ ^]^ Compared to these structures, which typically have dimensions similar to or smaller than the wavelength of visible light,^[^ ^58^ ^]^ the aerogel monoliths are several orders of magnitude larger and the question arises whether such macroscopic structures can be efficiently illuminated at all. The penetration depth of light into such large bodies is of fundamental importance, because if a large volume fraction of the photocatalyst cannot be illuminated, this part will not contribute to photocatalysis, thus reducing the yield per mass of the photocatalyst. Or in other words, if the penetration depth of the light is known, the geometry of the photocatalyst can be adjusted so that the entire monolith is efficiently irradiated and all the photoactive nanoparticles in the aerogel can participate in the reaction.

Little is known about the activity of monolithic aerogels compared to conventional photocatalysts already described in the literature. The most widely accepted value for comparing the activity of different photocatalysts is the apparent quantum yield (AQE). The AQE is calculated from the numerical ratio between the product(s) and the incident photons of a given wavelength in a reaction system.^[^ ^59^ ^]^ However, in order to compare aerogels with literature data, a suitable geometry of the aerogel monolith is needed as well as an appropriate reactor design.

**Figure 4:**
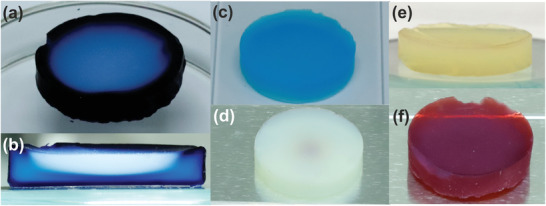
a) Top and b) side view photographs of a methyl blue impregnated titania aerogel monolith after several hours of irradiation with a 375 nm LED lamp. Photographs of a titania aerogel monolith with a low concentration of methyl blue c) before and d) after illumination for a few minutes. Photographs of a titania aerogel containing Au ions e) before and f) after illumination. Reproduced under the terms of the Creative Commons Attribution License CC BY NC 3‐0.^[^ ^59^ ^]^ Copyright 2021, The Authors, published by The Royal Society of Chemistry.

A simple and qualitative way to visualize the distribution of light in the monolith is to impregnate the aerogel monolith with a compound that, under the influence of light, is photocatalytically decomposed, resulting in a color change.^[^ ^59^ ^]^ A dye such as methyl blue is suitable for this purpose. Homogeneously distributed in a titanium oxide (anatase) aerogel, it degrades after irradiation with a 375 nm LED lamp, and the aerogel turns white at the areas reached by the light. The top view of such an aerogel after several hours of irradiation shows that exposure to the emitted photons results in a circular region with a much lower blue coloration (**Figure** **4**a). Of course, the degree and also the area of decolorization depends on the type of light source and its distance from the aerogel. The LED as a quasi‐point light source is not able to homogeneously irradiate the entire exposed top surface of the aerogel monolith. The light intensity at impact on the aerogel is highest in the center and decreases from the middle towards the edges. The next question is about the penetration depth of the light into the monolith, which can be illustrated by a cross‐sectional view of the irradiated aerogel. The white area in Figure 4b indicates the degradation of the dye inside the aerogel, proving that the photons can indeed reach the interior of the monolith. The semicircular shape of the illuminated area agrees well with the intensity distribution profile of the LED, and with an aerogel thickness of 3–4 mm, the penetration depth is about 1–2 mm. If the amount of dye is lowered (Figure 4c) then the aerogel monolith turns completely white (Figure 4d) even after just a few minutes of irradiation. Although the LED is a quasi‐point source, the light obviously diffuses throughout the monolith, but with decreasing intensity with respect to the photon‐rich region. In another experiment, gold ions were added to the titanium oxide aerogel. In this case, the photocatalytic reaction induces a reduction of the gold ions to gold nanoparticles, which changes the color of the aerogel from white (Figure 4e) to red (Figure 4f). The homogenous red color confirms that there is diffusive light transport in the aerogel monolith. For the photocatalytic hydrogen generation using methanol as feedstock, aerogels with Au only are not very efficient. Therefore, TiO_2_ aerogel monoliths were studied with Pd or PdAu nanoparticles in the same reactor (see Section 6) illuminated either from the top only or from two sides to increase the number of photons reaching the monolith. Indeed, it was found that the Pd‐TiO_2_ aerogel illuminated from two sides showed the highest H_2_ generation of 92 µmol h^–1^ with an apparent quantum efficiency of 13 % at a wavelength of 375 nm.^[^ ^59^ ^]^

The proper supply of light across the catalyst volume is an important element for achieving high efficiency in photocatalysis. However, a catalyst only develops its full potential if several gears mesh together accurately. Besides light transport into the catalyst, this also includes efficient photon absorption, charge generation, charge transport and transfer and, last but not least, sufficiently fast reactant supply to the active site.

## 4 Geometry and Mass Transport

While mass transfer in powders and films is generally easy to accomplish due to the short path lengths, the transport paths in 3D porous catalysts are several orders of magnitude longer. With the transition to macroscopic structures, questions inevitably arise about possible mass transfer limitations. Can the catalyst be supplied with sufficient reactants in a given time to make full use of the temporarily available charge carriers? Can the product be removed fast enough to avoid undesirable side reactions, blockage or even destruction of the microstructure? To answer such questions, it is essential to understand the characteristics and transport mechanisms relevant to mass transfer. Based on this information, a suitable geometry of the catalyst can be selected, and the reactor designed accordingly.

**Figure 5:**
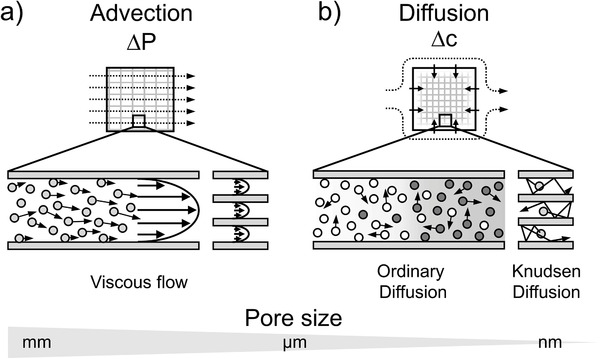
Prominent types of mass transport through a porous monolith. a) Advective flow driven by a pressure gradient Δ*P* is typically prevailing over diffusion in presence of large pores. The mass flow rate rapidly decreases for smaller pores. b) Diffusive mass transport along a concentration gradient Δ*c* dominates mass transport in pores of µm and smaller. Knudsen diffusion is a special type of diffusion and governs mass transport when molecule‐wall collisions predominate over molecule–molecule collisions. It is mainly relevant for gases. In case of intermediate pore sizes or materials with a broad pore size distribution, mass transfer occurs simultaneously via both mechanisms.

Mass transport in porous materials is typically divided into two categories: Advective and diffusive mass transport (**Figure** **5**).^[^ ^60^ ^]^ The proportion to which the two types of transport contribute to the overall mass transport depends to a significant extent on the pore size. Advection is a mass transport phenomenon based on a collective motion of molecules in a fluid caused by an external force. This type of flow plays an important role in pressure‐driven mass transport through porous media with pore sizes of several µm and larger. The flow rate scales with the pressure gradient and is also dependent on the viscosity of the fluid. Due to the much higher viscosity of liquids compared to gases, advective mass transport is about a hundred times slower for liquids than for gases.^[^ ^61^ ^]^ In addition to the fluid properties, the flow rate also greatly depends on the average pore diameter. If one moves from large pores to small pores, the viscous flow gets increasingly hindered due to the higher friction at the pore walls.

Below a certain pore size, mass transfer within the porous medium is mainly governed by diffusion processes. Mass transport via diffusion is the result of the thermal motion of individual molecules, which follow a random path through the media upon collision with other molecules or pore walls. In contrast to advection, the individual molecules move in a non‐directional manner. From a macroscopic perspective, however, the constant motion of molecules results in net flux along a concentration gradient, as on average more species diffuse from an area of high concentration to an area of low concentration than in the opposite direction. Diffusion rates are comparatively slow and depend to a large extent on the concentration gradient, but also on the molecular number density, molecular weight and temperature. Due to the high number density and the more pronounced interactions between the molecules, diffusion processes in liquids are about four to five orders of magnitude slower than in gases.^[^ ^61^ ^]^

Both advective and diffusive mass transport are much slower in liquids, making liquid‐phase reactions generally more susceptible to mass transport limitation compared to gas‐phase reactions.^[^ ^62^, ^63^ ^]^ Regardless of the medium used, the average pore size largely determines the extent to which advection or diffusion processes are involved in the overall mass transport. In larges pores, it is relatively easy to realize a rapid mass transfer by means of a pressure gradient. However, for materials with very small pores, efficient advective flow can no longer be achieved, so the overall transport rate is restricted to the rate of diffusion. Between these two extremes, i.e., in the case of intermediate pore sizes or broad pore size distribution, both mechanisms can be present to variable degrees. In practice, there are different aspects that need to be considered depending on the predominant type of transport, which will be briefly discussed in the following.

In pressure‐driven advective flow, the permeability of the porous material is a key characteristic that indicates how permeable a sample is to a particular fluid, or in other words, how fast mass can be transported through the porous medium due to a pressure gradient. The simplest way to determine this property experimentally is to apply a pressure gradient along an axis and measure the resulting volumetric flow rate. For this purpose, a uniform, fracture‐free sample with a defined macroscopic shape is necessary. Since permeability is directly proportional to the area and inversely proportional to the thickness of the sample, statements can be made about geometries suitable for a given setup. In the case of materials with large pores and a high permeability, the geometry usually plays a subordinate role when it comes to mass transfer limitation. In this case high flow rates can easily be achieved with low pressure losses, irrespective of the geometry. However, the situation is different if the permeability is low, which is often the case for catalysts with a large specific surface area and very small pores. In this case, an unfavorable geometry leads to high pressure differences along the sample even at low flow rates, which requires not only a more sophisticated reactor but also a high mechanical stability of the catalyst body, which is not always given, especially for highly porous materials. By choosing a plate‐ or disk‐like geometry, pressure losses can be kept within acceptable limits and material damage can be avoided. In this respect, volume‐penetrating cracks along the flow direction are a particular problem because they are usually much larger than the average pore size. Following the path of least resistance, a large part of the fluid passes through these channels, making uniform flow through the catalyst volume impossible. The situation is analogous with gaps located between the reactor wall and the catalyst body. Proper sealing is therefore essential to achieve flow through the body rather than around it. However, it is not always easy to find a suitable sealing that, on the one hand, provides good surface adhesion without penetrating the pore volume, and on the other hand, is compatible with the material, mechanically robust and chemically stable under operating conditions. On a laboratory scale, silicone rubber, epoxy glues or sealing waxes are often used for this purpose.

In contrast to catalysts with large pores, mass transport in materials with very small pores is mainly governed by diffusion processes. Whereas in the case of advection the feed to the catalyst can be easily controlled by adjusting the pressure gradient, the diffusion rate is much slower and at the same time greatly dependent on the concentration gradient. Diffusion rates are therefore difficult to manipulate, especially as the operating parameters such as fluid composition, pressure and temperature are usually preset. A high geometrical surface‐to‐volume ratio is crucial for efficient mass transfer in case of diffusion, as the reactants enter the volume exclusively via the outer surface of the catalyst body. A continuous feed stream over the catalyst is also beneficial, as it keeps the concentration gradients of product and educt at a high level. In heterogeneous catalysis, materials with small pore sizes are therefore often introduced into the reactor in the form of pellets for this purpose. The gaps between the pellets form a penetrating network of larger channels that allow efficient feed of reactants and removal of products by advective flow. Diffusion into the catalyst can take place over short paths, which significantly improves mass transfer. However, the illumination of such packed‐bed reactors can be challenging, because pellets with suitable optical properties are rare and the high number of fluid‐catalyst interfaces enhances reflection and scattering of the incident light.^[^ ^64^, ^65^ ^]^

Knowledge of the prevailing transport mechanisms is of great importance in order to take the right measures for optimizing mass transfer in a given system. Volume‐penetrating cracks, for example, can adversely affect mass transfer by pressure‐driven advection because they prevent uniform flow through the material. Conversely, such paths may even be desirable in a diffusion‐limited system, as they provide additional surface area leading to shorter diffusion pathways and thus improved mass transfer. However, the dominating transport mechanisms in porous media are not always easy to predict, especially when the inner structure is morphologically complex and difficult to characterize. Aerogels, for example, can exhibit a wide pore size distribution, ranging from subnanometer‐sized pores to pores of several hundred nanometers. For such a structure, the gas flow can be dominated by either advection or diffusion depending on the pore sizes present and operating conditions used.^[^ ^66^, ^67^, ^68^ ^]^ Deciphering such structures is often complex and requires a combination of different techniques such as electron microscopy, gas sorption analysis, X‐ray diffraction, light scattering, helium pycnometry or mercury porosimetry.^[^ ^69^ ^]^ The experimental study of transport mechanisms, on the other hand, typically requires macroscopic samples of well‐defined shape and high quality, which can be difficult to produce.

In a currently ongoing study in our group, we investigate pressure‐driven gas transport through nanoparticle‐based titania aerogels. The cm‐sized, carefully sealed samples exhibited remarkably low permeabilities despite a high porosity of up to 99 %. From the nitrogen gas sorption analysis, it was concluded that the low permeability is the result of the small pore sizes that are present in the material, ranging from only a few tens to a few hundreds of nanometers. Measurements at different average pressures and with different gases further revealed that the mass transport through the structure was mainly governed by Knudsen diffusion rather than advection. The driving force for diffusive flow in this case results from the applied pressure gradient, which in turn creates a concentration gradient between the two sides of the aerogel due to the compressibility of gases. Studies on samples with different densities and transparency have further shown that the permeability of nanoparticle‐based TiO_2_ aerogels can be controlled via the particle loading, while transparency has only a minor effect on the mass transfer rate. This independent tuning of light and mass transport offers great potential to improve the catalytic activity on the structural level. The knowledge gained about the primary transport mechanism, on the other hand, will enable the systematic selection of appropriate geometries and reactor designs to minimize mass transfer limitations and further improve the performance of titania aerogel catalysts in the future.

## 5 Geometry of the Monolith

Light and mass transport are two particularly critical factors in 3D photocatalysts, as we have seen in the previous discussion. To enhance the performance of such a photocatalyst, optimization not only at the material level but also at the geometry level is therefore inevitable. The choice of a suitable geometry depends strongly on the material properties (e.g., optical property and permeability), but also on the reactor geometry, the type of light source and the application.

The penetration depth of the light into the monolith is an important criterion in the choice of geometry. Ideally, the sample thickness is adjusted in a way, that the incident light is fully utilized without leaving a significant part of the catalyst volume unilluminated. In certain cases, smaller sample thicknesses can be advantageous, especially when uniform light distribution are important and efficient light utilization is of minor importance. This may be the case, for example, when studying mass transfer limitations or photocatalytic activities in an early stage of research. On an industrially relevant scale, however, the optimal use of available light is of central importance. The absorption coefficient, and thus the penetration depth, scales with the solid volume fraction.^[^ ^70^ ^]^ In case of dense and porous titania films, layer thicknesses of a few µm are typically sufficient for complete absorption of UV light.^[^ ^71^, ^72^ ^]^ For very low‐density monolithic titania aerogels, however, the penetration depth of light is in the mm range and relatively thick layers of materials are required for adequate light absorption, which in turn renders mass transfer more difficult.^[^ ^59^, ^73^ ^]^

To achieve fast mass transfer, thin samples are generally advantageous, regardless of the predominant type of mass transfer (i.e., advection or diffusion). However, statements about optimal thickness from this point of view are less trivial. In pressure‐driven advection through permeable monoliths, pressure losses are an important variable, which can be easily adjusted by choosing a suitable monolith thickness and cross‐section. High pressure losses are particularly problematic in applications where high flow rates are required, for example in air purification.^[^ ^62^ ^]^ In diffusive mass transfer, shorter diffusion paths lead to an improved reactant supply. At the same time, however, the residence time of the reactants and products in the catalyst decreases, which can have an adverse effect on selectivity and yield. The extent to which such considerations are relevant depends strongly on the desired application.

**Figure 6:**
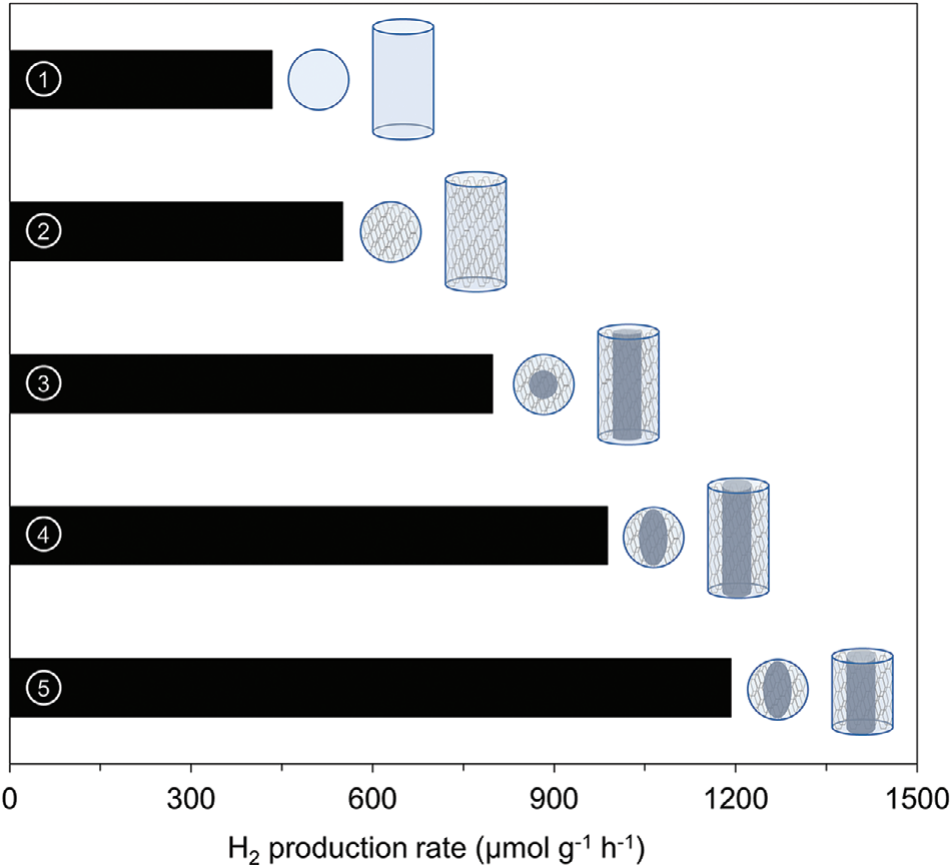
Hydrogen production rate of TiO_2_‐Pd aerogels synthesized with the aid of various scaffold geometries: 1) no scaffold, 2) standard scaffold, scaffold with solid core with 3) circular cross‐section, scaffold with solid core with 4) elliptical cross‐section, scaffold with 5) solid core with elliptical cross‐section and optimized length. Reproduced under the term of the Creative Commons Attribution Licence CC BY‐NC 4.0.^[^ ^73^ ^]^ Copyright 2021, The Authors, published by Wiley‐VCH.

The geometry of aerogel catalysts can in principle be chosen simply by selecting a suitable mold for the gelation. However, if one wants to vary the structure inside the monolith in addition to the outer shape, the whole endeavor becomes very complicated. Cylindrical monoliths represent one of the simplest and most widespread shapes and seem to be ideally suited for use in a tubular flow reactor. In such a reactor, the gas flows in the direction of the cylinder axis, while the illumination occurs from the side. This orthogonality of light and gas flow, however, introduces some inherent problems, especially when the penetration of light into the catalyst and the mass transport through it are limited. To ensure good contact between reactants and catalyst, a cylindrical shape with a large cross‐section and low thickness would be desired. At the same time, however, such a geometry only offers a small lateral area for illumination, which makes light supply more difficult to realize. An obvious way to tackle this apparent dilemma seems to be the use of small beads instead of monolithic bodies.^[^ ^74^, ^75^ ^]^ Unfortunately, it is difficult to produce unsupported granules of suitable optical quality, so that the improved mass transfer in packed columns comes at the expense of adequate illumination. An alternative approach to improve mass and light transport is the use of scaffolds to fit the catalyst geometry to a particular reactor design. In a recent study, carefully designed 3D printed scaffolds were introduced into cylindrical TiO_2_‐Pd aerogel monoliths to optimize their photocatalytic performance in a tubular reactor.^[^ ^73^ ^]^ It is important to note that in this case, the embedded scaffold did not serve as a support for the photocatalyst, but rather as a geometric constraint. The photocatalyst itself formed a volume‐filling and self‐supporting network in the cavities of the scaffold. **Figure** **6** summarizes the different scaffold geometries with the corresponding hydrogen production rates during methanol reforming under UV light.

he introduction of a scaffold not only increases the mechanical stability, which significantly facilitates the handling and mounting of the brittle aerogels into the reactor, but also leads to an increase in the absolute production rate compared to the conventional monolith. The reason for the higher efficiency was attributed to volume‐penetrating gaps that form at the interfaces between the photocatalyst and the polymer scaffold during sample preparation. Such pathways enable advective mass transport through the center of the monolith and significantly shorten diffusion‐dominated mass transport into the catalyst volume. Further improvement of reactant supply could be achieved by introducing a perforated hollow tube in the center of the aerogel. Apart from optimizing mass transfer, such polymer scaffolds also enable systematic adjustments of the geometry with respect to light irradiation. In this case, illumination was provided by a commercially available LED light point source, which results in inhomogeneous illumination in the axial and lateral dimensions of the monolith. By adjusting its length and matching the layer thickness to the light penetration depth, the inefficiently irradiated catalyst volume could be reduced, resulting in a three times higher hydrogen production rate per catalyst mass compared to the conventional monolith.

Studies such as this illustrate the extent to which photocatalytic performance can be improved by simply adapting the catalyst geometry to the reactor design. Unlike molds, which primarily dictate the external macroscopic shape, 3D printed scaffolds can also create structuring within the interior of the monolith. We see great potential in geometry optimization through direct 3D printing of photoactive materials. In addition, the fabrication of geometries that can be used in packed‐bed reactors is also a promising approach that we are currently pursuing. Matching geometry of the catalyst to the reactor design is one direction that can be taken to optimize the photocatalytic performance. Equally important, however, is the adaptation of the reactor design to the materials properties and the available geometries. Many different reactor designs exist, some of which will be discussed in the following.

## 6 Geometry and Reactor Design

Optimizing the geometry of the photocatalyst only makes sense if it is perfectly matched to the reactor. Geometry and reactor design are closely linked and must be considered together. Both the gas supply system and the illumination must be taken into account in the design of the reactor, since both are affected by the geometry of the monolith. We have already discussed how illumination and geometry are related in Section 3, and we have also addressed geometry and mass transport in Section 4. Of course, it is advantageous if the photoreactor design is as flexible as possible and covers many different experimental parameters, such as the possibility to use and compare monoliths as well as films and powders, to vary the reaction temperature, wavelength and light source, or to investigate gas mixtures, different pressures and different flow rates.

Although the majority of publications in the field of photocatalysis is devoted to materials development, many different photoreactors have been reported.^[^ ^76^, ^77^, ^78^, ^79^ ^]^ Photocatalytic reactors are generally classified according to how the photocatalyst is present in the reactor, either as a slurry or immobilized on a solid surface.^[^ ^32^, ^80^ ^]^ For batch‐operated liquid‐phase processes (e.g., water purification), slurry photoreactors are most common, although the often complicated separation of the photocatalyst reduces its reusability and also the post‐reaction separation of the products can be very energy intensive. Nanoparticles, which are extensively studied as photocatalysts due to their large surface area, are particularly difficult to separate. Such problems can be avoided by immobilizing the photocatalyst on a solid support. This approach is very common in flow reactors. It has the added advantage that the geometry of the substrate can be adjusted by introducing channels to optimize the flow and keep the pressure drop low. At the same time, there is some risk of catalyst loss, as catalyst particles may detach and be carried away by the flow (be it gas or liquid). Moreover, the exposed surface per unit mass is lower compared to slurry reactors, and efficient and homogeneous irradiation of the entire photocatalyst is difficult.

**Figure 7:**
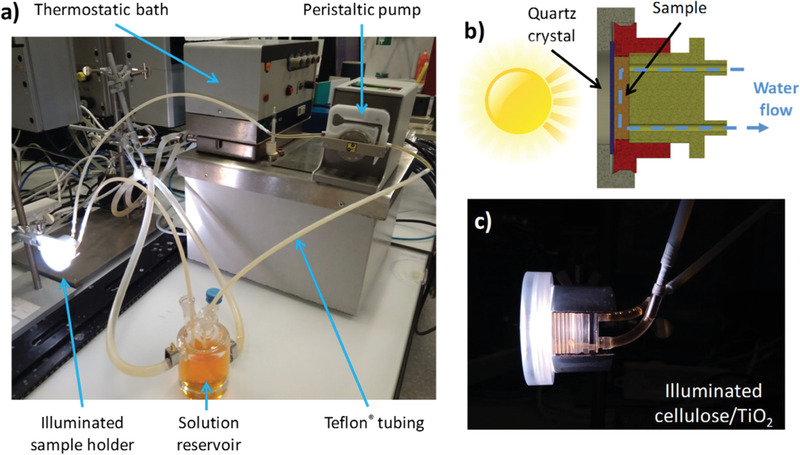
a) Digital photograph of the experimental setup used for the photocatalytic tests with the orange solution of the pollutant in water, b) sketch of the customized sample holder for the cellulose‐titania hybrid monolith with the water flow through the monolith (labelled with sample) and c) a digital photograph of the illuminated sample holder. Reproduced with permission.^[^ ^88^ ^]^ Copyright 2018, American Chemical Society.

There is not much literature about photoreactors for monolithic photocatalysts of macroscopic size. Most of them were developed for the decomposition of organic pollutants in air or in water, and all of them belong to the category of supported/immobilized photocatalysts, i.e., the photocatalytically active material is coated as a thin film of a few µm on a carefully designed 3D solid substrate. While the geometry of the substrate can be chosen to maximize mass transfer, efficient illumination of the interior of the monolith remains challenging. Ceramic monoliths with honeycomb channels that are coated with TiO_2_ are the most popular systems. While Sauer and Ollis illuminated such a monolith with light sources from the outside,^[^ ^81^ ^]^ indeed facing problems with the light intensity inside the channels, Valsaraj et al. solved that by running optical fibers through the monolith. They used a ceramic monolith with 61 honeycomb cylindrical channels, which provide a good surface‐to‐volume ratio and allow high flow rates with low pressure drop.^[^ ^82^ ^]^ Quartz fibers were inserted into each channel to act as light transmitters. The optical fibers were also coated with TiO_2_ to allow UV light to exit the fibers radially, but the coating was thinner than that on the ceramic substrate. With a length of 30 cm and with a channel diameter of 3 mm the monolith represented a truly macroscopic and 3D structure. Although such a design is elegant, several challenges remain. The volume of the reactor is not used efficiently. A large part of the photocatalyst consists of support material, which does not contribute to the photocatalytic reaction. The adhesion of TiO_2_ to the glass fibers is not very strong, which affects the durability, and it was found that the propagation length of light in the glass fibers is only a few cm.^[^ ^82^ ^]^ Therefore, this reactor design was improved by using non‐TiO_2_ coated quartz fibers with efficient side light emission over a fiber length of 35 cm.^[^ ^83^ ^]^ Other ways to mitigate the illumination problem include the use of transparent support materials such as glass on which the photocatalyst is deposited, or the use of photocatalysts in the form of thin membranes in which the photocatalyst is immobilized on a ceramic or polymer membrane.^[^ ^84^, ^85^, ^86^ ^]^ The type of membrane determines its mechanical and chemical stability, wettability, and tendency to foul. For glass supports it is difficult to fabricate complex 3D structures, and the support geometry is rather limited to simple shapes like spheres and platelets.^[^ ^87^ ^]^ For membrane photocatalysts, it is advantageous for efficient irradiation to have a thin membrane with high porosity (i.e., low density), but on the other hand this reduces the contact area between the photocatalyst and the substrate and also the residence time of the reactant in the reactor. All these issues can be addressed by using a titania‐cellulose hybrid monolith for in‐flow purification of water under solar light.^[^ ^88^ ^]^ The hybrid monolith has a high porosity of 98 %, a surface area of 80 m^2^ g^−1^, and the organic scaffold provide a highly flexible and mechanically stable 3D structure. Using a custom‐made continuous flow reactor (**Figure** **7**a) with a holder that guides the aqueous solution through the monolith (Figure 7b,c), a good photocatalytic activity and a long‐term stability of the hybrid monolith toward the decomposition of methyl orange and paracetamol was observed under artificial sunlight.

In addition to air and water purification, photocatalytic CO_2_ reduction has gained tremendous importance in recent years, prompting scientists to develop reactors also for this reaction. There are two key parameters that determine the types of photoreactors for CO_2_ reduction, namely the phases involved and the mode of operation.^[^ ^87^ ^]^ The phases include gas‐solid, liquid‐solid or gas‐liquid‐solid, while the mode of operation refers to batch, semi‐batch or continuous. Similar to pollutant degradation, the photoreactors for CO_2_ reduction can be divided into the two main categories of suspended/slurry reactors and immobilized/fixed bed systems, and also the reactor design for the study of CO_2_ reduction in the gas phase is quite similar: The photocatalyst powders are dispersed as a thin layer on a flat substrate for batch experiments or packed in a tubular quartz capillary for flow reactors.^[^ ^89^ ^]^ If the substrate is thermally conductive, then also heat energy can be supplied to the photocatalyst to drive a photothermal reaction.^[^ ^90^ ^]^

**Figure 8:**
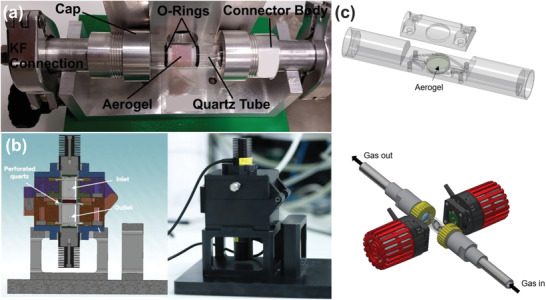
a) Photograph of a continuous flow photoreactor for cylindrical aerogels (here TiO_2_‐Au) inside a quartz tube illuminated with a solar simulator from the backside. Reproduced with permission.^[^ ^54^ ^]^ Copyright 2017, Royal Society of Chemistry. b) Schematic (left) and photograph of a flow reactor for plate/disk‐shaped aerogel monoliths that can be illuminated from two sides by LEDs. Light and gas flow are parallel. This reactor can also be heated. Reproduced under the terms of the Creative Commons Attribution License CC BY‐NC 3‐0.^[^ ^59^ ^]^ Copyright 2021, The Authors, published by The Royal Society of Chemistry. c) Sketch of a flow photoreactor for plate/disk‐shaped aerogel monoliths with light and gas flow in perpendicular direction. Top image shows the sample holder for the aerogel monolith, while the bottom image shows the whole reactor system with gas in‐ and outlet and with the two LEDs (in red). Reproducedunder the terms of the Creative Commons Attribution License CC BY‐NC‐ND.^[^ ^91^ ^]^ Copyright 2021, The Authors, Published by American Chemical Society.

In contrast to all the examples discussed so far in Section 6, we now turn to nanoparticle‐based aerogels as macroscopic photocatalysts that are completely self‐supporting, i.e., there is no 3D support that dictates the geometry of the photocatalyst. This has the great advantage that no material other than the photocatalyst absorbs the light, and much higher catalyst loadings per volume can be realized allowing very compact device design. At the same time, however, the demands on the monoliths and thus on their manufacturing process are extremely high. Only if they can assume a defined geometry that is large enough and also mechanically stable enough for handling and mounting it makes sense to develop specific reactors for them. Although the mechanical stability of the aerogels can be improved by optimizing the manufacturing process, their fragility remains a major challenge in reactor development. Just imagine developing a reactor for a cylindrical monolith, where the monolith breaks into smaller pieces when it is introduced into the reactor. Any advantage of the defined macroscopic geometry would be immediately lost. The first photoreactor developed in our group was indeed fabricated for cylindrical monoliths (Figure 3b, TiO_2_ or TiO_2_‐Au) with dimensions of about 1 cm in diameter and 1 cm in height.^[^ ^54^ ^]^ It was designed as a flow reactor to study the photoreduction of CO_2_ in the gas phase with water vapor. **Figure** **8**a shows the reactor system with two KF connectors attached to the quartz tube with the aerogel fixed inside with Teflon centering rings and NBR O‐rings to guide the gas into the monolith, minimizing its flow along the quartz tube – monolith interface. The reactor inlet is connected to the gas preparation setup, where CO_2_ gas was bubbled through water to enrich it with humidity. The flow rate was controlled by a mass flow meter. A Xe lamp irradiated the monolith from one side through a circular whole in the metal plate. The outlet stream was analyzed by mass spectrometry and gas chromatography. Similar to all the photocatalytic reactors discussed above, our reactor suffered from the same issues: Photon and mass transfer limitations. Although the aerogel monoliths are translucent, the penetration depth of light, especially if it comes from only one side, is not sufficient for such cylindrical monolith dimensions. Moreover, such a tubular design turned out not to be ideal for monoliths with a low gas permeability. While at very low gas flow rates the pressure drop across the aerogel is negligible, it becomes problematic at higher flow rates as the seal is not effective enough to withstand the high pressure gradients allowing the gas to bypass the sample (see Section 4). There are two ways to address these problems of light and gas supply. One is to keep the macroscopic shape of a cylinder but adjust the internal geometry, and the other is to change the shape of the monolith into a platelet or a disk. For the first strategy the reactor does not need to be fundamentally changed. The use of tailor‐made scaffolds with cylindrical macroscopic shape, but with varying internal structure to modify the gas flow, and thus the mass transfer, and to increase the illumination efficiency was explained in Section 5.^[^ ^73^ ^]^ For the second option, when the geometry is changed from cylinder to disk, also the design of the reactor has to be modified. We developed two new reactors that illuminate the disk both from above and below (i.e., from the flat sides), while allowing the gas to flow either in the same direction as the light (along the axis of the disc) as shown in Figure 8b or perpendicular to the light, i.e., along the cross‐section of the disk (Figure 8c). Two‐sided illumination is more efficient in any case and for all three reactors, LEDs are usually preferred over artificial sunlight because they are more compact and the wavelength and power can be selected very conveniently. The preferred orientation of the disk in the gas stream depends on the predominant type of mass transport within the sample. An arrangement as shown in Figure 8b is suitable if the structure of the monolith allows mass transfer by advection. By using thin disks, high pressure losses and associated mechanical stresses can be avoided, and the required sealing is easy to implement. In contrast, the configuration shown in Figure 8c is more suitable for samples where mass transfer into the catalyst volume is restricted to diffusion. The continuous stream over the sample ensures high concentration gradients and optimized contact between reactants and monolith. In all the examples given above, the gas mixing and gas analysis systems are basically independent of the reactor design. Both reactors were investigated for gas‐phase methanol reforming using Pd‐, PdAu‐TiO_2_^[^ ^59^ ^]^ and N‐doped TiO_2_ with Pd.^[^ ^91^ ^]^

When developing a photoreactor for monoliths, many parameters must be taken into account. Complexity plays a role in the design, as does cost. Repair possibilities, spare parts and gas tightness are also included here. An important part of reactor design is illumination, and there is the question of how much of the light can really be used by the photocatalyst and how to quantify that. For efficient mass transport, good contact between reactant and photocatalyst must be ensured, which is supported by a large surface‐to‐volume ratio. An important aspect of reactor design is the reactor volume, which of course directly determines the size of the monolith, but also influences the establishment of possible chemical equilibria or the length of the mass transport paths. Last but not least, options such as the ability to change the temperature and study different monolith shapes are beneficial to cover as many experimental parameters as possible. The different reactors discussed in Section 6 differ in many of these aspects. The tubular flow reactor in Figure 8a is inexpensive, and its simple design makes it easy to locate and fix leak points. However, illumination is inhomogeneous and orthogonal to the gas flow, and the reactor cannot be heated. The reactor in Figure 8b is more complex which makes leak detection and cleaning much more tedious, but illumination is more efficient and homogeneous, the light can be quantified, and the direction of the gas flow and the light is the same. Heating is possible and, certainly a big advantage, the reactor works for samples with different geometries, including monoliths, powders and films. This geometric flexibility comes at the price of a large internal volume, that is less suitable for low flow experiments and requires longer purging times. The design of the reactor in Figure 8c is similar to the one in Figure 8a, but thanks to the different orientation of the monolith the illumination is more homogeneous and the contact between reactant and photocatalyst is improved.

The preceding discussion shows that the development of a photoreactor is a complex undertaking. Although the reactor is a critical element in the development of photocatalysts, there is no standard solution that can be applied. Instead, the design depends on many different factors, including material properties, so researchers often have to rely on tailor‐made reactors. Many groups that specialize in the synthesis of new photocatalysts are not necessarily also experts in reactor design. In general, it is rather difficult to cover all aspects of material synthesis, reaction control, product analysis and reactor design in a single research group. This is why increased efforts are needed to work across disciplines and bring together chemists, materials scientists as well as engineers to explore new concepts in photocatalysis such as the use of macroscopic 3D photocatalysts in their full range and explore their potential as much as possible in applications.

## 7 Conclusion

Among the various ways to improve the photocatalytic efficiency of a system, optimization of the macroscopic geometry of the photocatalyst has received little attention. However, we know from classical catalysis that this aspect plays a prominent role. The macroscopic geometry allows the surface area and mass transport to be tuned and the overall throughput to be increased, both of which are extremely important for technological upscaling. These parameters and considerations are also important in photocatalysis, but compared to classical catalysis, the constraint of having to additionally illuminate the catalyst makes the development of such systems more difficult. On the photocatalyst side, there are not many examples where macroscopic architectures are produced and applied. In most cases, these are surface‐immobilized materials, with the opaque substrate posing another challenge for illumination. Self‐supporting transparent photocatalysts such as aerogels may be an attractive alternative here. They can be selectively assembled from a wide variety of nanoparticles and manufactured in different geometries. By using scaffolds, the geometric complexity, both in terms of external and internal shape, can be significantly increased in order to optimize both illumination and mass transport. To really get the most out of a photocatalytic system in the end, the macroscopic shape of the photocatalyst must be precisely matched to the geometry of the reactor. It is not possible to develop one of the two components independently of the other. In this review, we have discussed what geometric factors in particular need to be considered in the fabrication of macroscopically large monolithic photocatalysts to enable efficient illumination and fast mass transport, and how these factors affect photoreactor design. We are convinced that the combination of monolithic photocatalysts with tailor‐made photoreactors is an interesting concept, which not only opens new horizons for basic research, but also represents a great potential for the technological use of such processes.

## Conflict of Interest

The authors declare no conflict of interest.
